# A Comprehensive Phylogenetic and Bioinformatics Survey of Lectins in the Fungal Kingdom

**DOI:** 10.3390/jof7060453

**Published:** 2021-06-07

**Authors:** Annie Lebreton, François Bonnardel, Yu-Cheng Dai, Anne Imberty, Francis M. Martin, Frédérique Lisacek

**Affiliations:** 1 Beijing Advanced Innovation Center for Tree Breeding by Molecular Design, Beijing Forestry University, Beijing 100083, China; annie.lebreton@inrae.fr (A.L.); yuchengdai@bjfu.edu.cn (Y.-C.D.); 2 University of Grenoble-Alpes, CNRS, CERMAV, 38000 Grenoble, France; francois.bonnardel@cermav.cnrs.fr; 3 Swiss Institute of Bioinformatics, CH-1227 Geneva, Switzerland; 4 Computer Science Department, UniGe, CH-1227 Geneva, Switzerland; 5 Université de Lorraine, INRAE, UMR Interactions Arbres/Microorganismes (IAM), Laboratoire d’Excellence ARBRE, Centre INRAE GrandEst-Nancy, 54280 Champenoux, France; 6 Section of Biology, UniGe, CH-1205 Geneva, Switzerland

**Keywords:** lectins, MycoCosm, carbohydrate recognition

## Abstract

Fungal lectins are a large family of carbohydrate-binding proteins with no enzymatic activity. They play fundamental biological roles in the interactions of fungi with their environment and are found in many different species across the fungal kingdom. In particular, their contribution to defense against feeders has been emphasized, and when secreted, lectins may be involved in the recognition of bacteria, fungal competitors and specific host plants. Carbohydrate specificities and quaternary structures vary widely, but evidence for an evolutionary relationship within the different classes of fungal lectins is supported by a high degree of amino acid sequence identity. The UniLectin3D database contains 194 fungal lectin 3D structures, of which 129 are characterized with a carbohydrate ligand. Using the UniLectin3D lectin classification system, 109 lectin sequence motifs were defined to screen 1223 species deposited in the genomic portal MycoCosm of the Joint Genome Institute. The resulting 33,485 putative lectin sequences are organized in MycoLec, a publicly available and searchable database. These results shed light on the evolution of the lectin gene families in fungi.

## 1. Introduction

Fungi are unicellular or multicellular organisms found in terrestrial, marine and aquatic ecosystems. They adopt a wide range of ecological lifestyles (e.g., modes of nutrition) along the saprotrophism/mutualism/parasitism continuum [1,2,3]. Saprotrophic, symbiotic and pathogenic fungal species compete or cooperate with multiple bacteria, fungal, plant or animal species. Specific signaling and developmental pathways underpin the establishment and maintenance of interactions with other organisms. Self/non-self-discrimination is at the very core of the fungal hyphae development and involves surface-surface molecular interactions [4]. Similarly, the recognition of animal or plant surfaces play a key role in the pathogenic or mutualistic fungal symbioses. These interactions rely on a wide range of secreted proteins that include lectins.

Lectins are ubiquitous carbohydrate-binding proteins playing a crucial role in self/non-self-recognition through specific interactions with complex glycans present on the surface of proteins, microorganisms or tissues [5]. These small proteins are therefore involved in a wide range of biological processes such as reproduction and development as well as establishment of biotrophic associations but also in many pathogenesis-related mechanisms, such as host–pathogen interactions and tumor metastasis. A large number of lectins have been identified in filamentous fungi and yeasts [6,7,8,9,10]. They are generally considered as defense-related proteins. This role is well documented in mushroom-forming fungi, where lectins are known to protect these reproductive structures from hyphal grazers, such as nematodes, slugs, snails and insects, through their nematotoxic and entomotoxic activities [11].

Except for their role in defense, the current body of information on the biological and ecological roles of lectins in saprotrophic, mutualistic and pathogenic fungi is scarce. Some yeasts produce flocculins that play a role in the formation of multicellular structures of interest for brewers and winemakers [12,13]. Pathogenic yeasts, such as *Candida albicans*, rely on adhesins to initiate the colonization of epithelial hosts [14], while filamentous pathogenic fungi, such as *Aspergillus fumigatus*, produce soluble lectins involved in host recognition and are able to trigger signaling pathways leading to lung inflammation [15,16]. Less is known about the role of lectins in the establishment of fungal–plant or fungal–animal mutualistic symbioses. Fungal lectins were suggested to contribute to the mutualistic ectomycorrhizal symbiosis between the basidiomycete *Lactarius deterrimus* and its host tree, *Picea abies* [17], but this is not yet confirmed by genetic disruption of the corresponding genes. A galactose-specific galectin was shown to act as a chemoattractant for the recruitment and adhesion of the cyanobacterial *Nostoc* symbiont to the hyphae of *Peltigera canina*, leading to a lichen symbiosis [18]. A large number of lichen associations are now confirmed [19].

Lectins play crucial roles in fungi, but their unique properties for specific binding to complex sugars make them highly relevant tools in analytical glycomics, biochemistry and immunology [20]. Fungal lectins are of potential interest in agriculture as entomotoxic and nematotoxic compounds [11]. Some fungal lectins specifically bind oligosaccharide epitopes on cancer cells [21,22] and are therefore promising tools for diagnosis of drug release. These examples emphasize the relevance of discovering new lectins in the fungal kingdom. 

In the genomic era, a very common discovery strategy entails in silico sequence analysis. However, information extraction from protein and genome sequence databases is significantly hampered by the poor biochemical and functional knowledge of fungal lectins, resulting in low-quality annotation. Furthermore, 3D structural data are still scarce and do not reflect the variety described, for example in a recent review reporting 26 fungal lectins displaying 10 different folds in 100 crystal structures [23]. Using the hierarchical 3D-fold based classification of the curated UniLectin3D database (www.unilectin.eu/unilectin3D) [24], conserved sequence motifs were identified in each of the 109 lectin classes. The classification defined in UniLectin3D applies to collected lectins across all living kingdoms. The conserved motifs are characteristic profiles that can be used to map the lectin repertoire, called lectome, of a given species. With this approach, the lectin repertoire of thousands of eukaryotic, bacterial and archaebacterial species was generated and stored in LectomeXplore, another module of the UniLectin platform [25]. In the present study, the objective was to adopt this strategy with a special focus on Fungi and to accurately predict and classify lectins. To that end, we used the UniLectin3D-based motifs to screen fungal genomes available at the Joint Genome Institute (JGI) MycoCosm genomics portal (http://jgi.doe.gov/fungi) [26]. This database collects genomes representative of most fungal lifestyles and supports the integration, analysis and dissemination of fungal genome sequences as well as other “omics” data through interactive web-based tools [26]. In the 2020 release of this database used in the present study, approximately 1419 genomes of 1223 species were stored. Sequence processing resulted in the identification of 35,460 lectin domains in 33,485 putative lectins. These predictions can be searched and analyzed in the MycoLec module of UniLectin (https://www.unilectin.eu/mycolec/). 

The potential impact of the mode of nutrition and lifestyles on the lectin gene repertoire was investigated in the fungal species inhabiting forest ecosystems. A large part of the fungal species whose genome is available at the JGI MycoCosm and NCBI databases populates these terrestrial ecosystems. They include specialized saprotrophs (litter/soil decayer, wood decayer, etc.) and symbiotrophs (e.g., ectomycorrhizal fungi), with most species belonging to the Agaricomycetes class. To reduce the phylogenetic signal observed in the lectin diversity and to assess the impact of ecology and mode of nutrition on the lectin content and distribution, we narrowed this study to the Agaricomycetes. In this article, we first present the results of the comprehensive mapping of the different groups of lectin genes and the examination of the origin and evolution of these gene families in Fungi. Then, we focus on Agaricomycetes in order to single a few lectin candidates out of the initial thousands to be further characterized with functional analyses.

## 2. Materials and Methods

### 2.1. Fungal Lectin Classes

Details of the lectin classification are given in [25]. Briefly, the domain sequences were extracted from the three-dimensional lectin structures available in UniLectin3D [24] and lectin fold sharing more than 20 percent sequence similarity were grouped in the same class. This classification applied to 3D structures of fungal lectins.

### 2.2. Fungal Genomes

The 1223 fungal proteomes of the 2020 release of the MycoCosm platform (mycocosm.jgi.doe.gov) [26,27] were downloaded. The full taxonomy of each corresponding fungal strain was retrieved using the ncbitax2lin software tool (github.com/zyxue/ncbitax2lin). When ncbitax2lin did not identify the full taxonomy of the fungal strains, taxonomy was assigned manually with the support of JGI (taxonomy.jgi-psf.org) and UniProt (www.uniprot.org/taxonomy). The ecological guild of all fungal strains was automatically annotated using FUNGuild [28], and expert ecological annotation was performed for the 125 Agaricomycetes strains available at the time of this study.

### 2.3. Identification and Scoring of Candidate Fungal Lectins

For each class, the lectin 3D domain sequences from which histidine tags were removed were aligned with MUSCLE [29]. The obtained alignments were used to generate a Hidden Markov Model (HMM) for each domain. HMMSEARCH [30] was then run to identify lectins in the dataset, with a e-value threshold at 10^−2^. Results were uploaded in the MycoLec database to provide direct web access to the identified lectin candidates. Lectins were further filtered by a score, reflecting sequence similarity between the reference lectin domain consensus sequence and the predicted lectin domain sequence [25]. This score was computed using the amino acid sequence alignment generated by HMMER during the search. At each position of the alignment, a cumulative counter was incremented by 1 if amino acids were identical, else by a normalized BLOSUM62 substitution score. The final value of the counter divided by the domain length (i.e., the total number of positions) resulted in a value between 0 to 1 that defined this similarity score. This score was also used to rank lectin candidates on the web display of MycoLec content.

### 2.4. Transcriptomics

Differentially expressed genes of 14 fungal–plant interactions leading to mycorrhizae formation were collected from the literature (detailed in Table S1). In this published work, the authors compared the expression of fungal genes between established mycorrhizae stage with a compatible plant and free-living mycelia (control condition) in vitro [28,31,32,33]. In all studies, genes were considered as differentially expressed during mycorrhization compared with control when a log2 fold change above 2 or below −2 and an adjusted *p*-value below 0.05 were observed. Among the fungal strains, three are ericoid mycorrhizae (ERM), two are orchids mycorrhizae (ORM) and the remaining are ectomycorrhizae (ECM) (Table S1). All 14 fungal strains pertained to our genomic dataset.

## 3. Results

### 3.1. Structural Classification of Fungal Lectins in UniLectin3D

**Figure 1:**
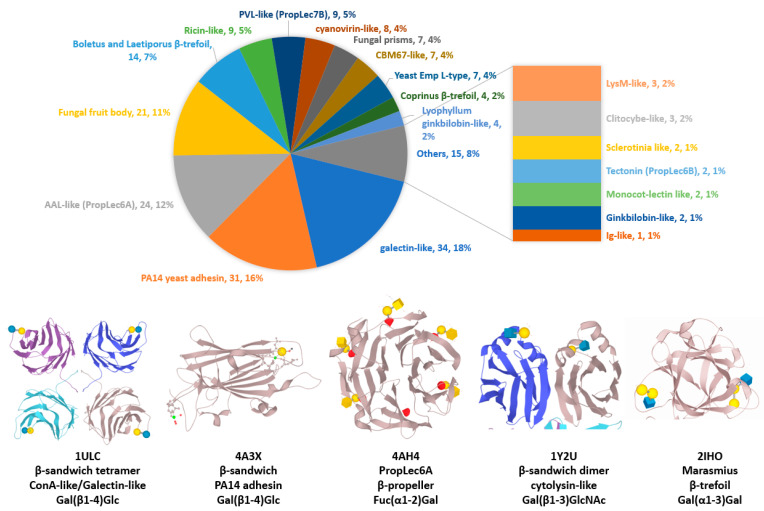
Distribution of fungal lectins 3D structures classes in UniLectin3D accessed on January 2021. Bottom: 3D view of most common folds for fungal lectins, using selected structures from the PDB (www.rcsb.org) [35]: 1ULC from *Coprinopsis cinerea*; 4A3X from *Candida glabrata;* 4AH4 from *Aspergillus fumigatus*; 2IHO from *Marasmius oreades*; 1Y2U from *Agaricus bisporus.*.

The 2021 version of the Unilectin3D database contains 549 distinct lectin sequences and 2278 lectin 3D structures, including 1456 with their interacting ligands [24]. Among these lectins, 194 structures belong to fungal species. The known 3D structures of fungal lectins are partitioned in 12 different folds (for a total of 35 in the database), expanding into 20 classes (for a total of 109 in the database) (Figure 1 and Figure S1). Except for the ß-prism III fold, identified fungal lectin folds are shared with other organisms. The most abundant lectin 3D structures in the surveyed fungi are the ß-sandwich/Con-A-like fold (24%), the ß-propeller fold (18%) and the ß-trefoil fold (17%). When looking at classes, some are very specific of fungi, for example fungal fruit body lectins. The most represented classes include galectin-like (18%), PA14 yeast adhesin (15%), AAL-like (12%), fungal fruit body lectin (11%) and *Boletus* and *Laetiporus* ß-trefoil lectin (7%) (Figure 1). While lectins are often forming multivalent protein complexes through monomer oligomerization, several fungal lectins generate multivalent binding sites through internal repeats, as in the case of ß-trefoils and ß-propellers [34].

### 3.2. The MycoLec Database of Fungal Lectomes

**Figure 2:**
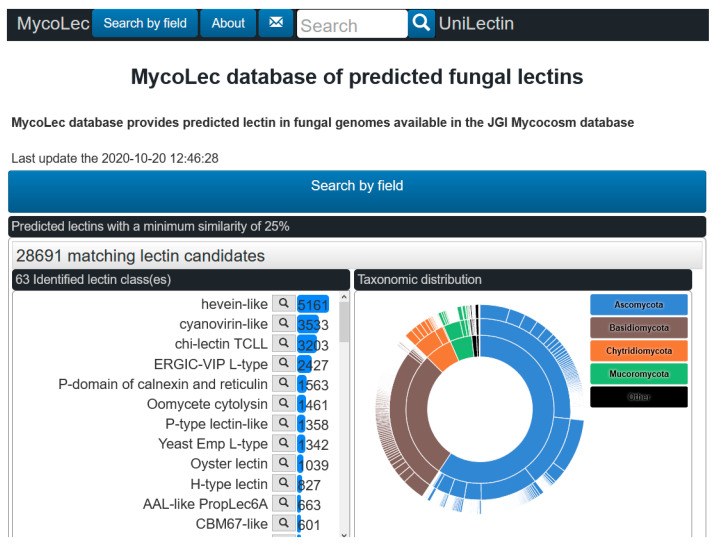
Access page of the MycoLec database with information about the distribution of fungal lectins as a function of classes and as a function of the fungal taxonomy.

The MycoLec database (https://www.unilectin.eu/mycolec/) shares the same architecture as the LectomeXplore database and is dedicated to Fungi. The prediction from the 1419 translated fungal genomes of the Mycocosm portal (February 2020 release) resulted in approximately 28,700 sequences of putative lectins with a score greater than 0.25. The entry page of the database is displayed in Figure 2, showing the different entry points: lectins can be accessed by clicking on the different classes (left panel, with number of sequences indicated for each class), or on a taxonomy sunburst (right panel). Alternatively, the “search by field button” enables the combination of search terms such as, genus, species, classes or selected keywords. Each candidate lectin is associated with sequence information including the alignment with the reference sequence of the predicted lectin class.

### 3.3. Identification of Lectin Domains in Fungal Proteomes

**Figure 3:**
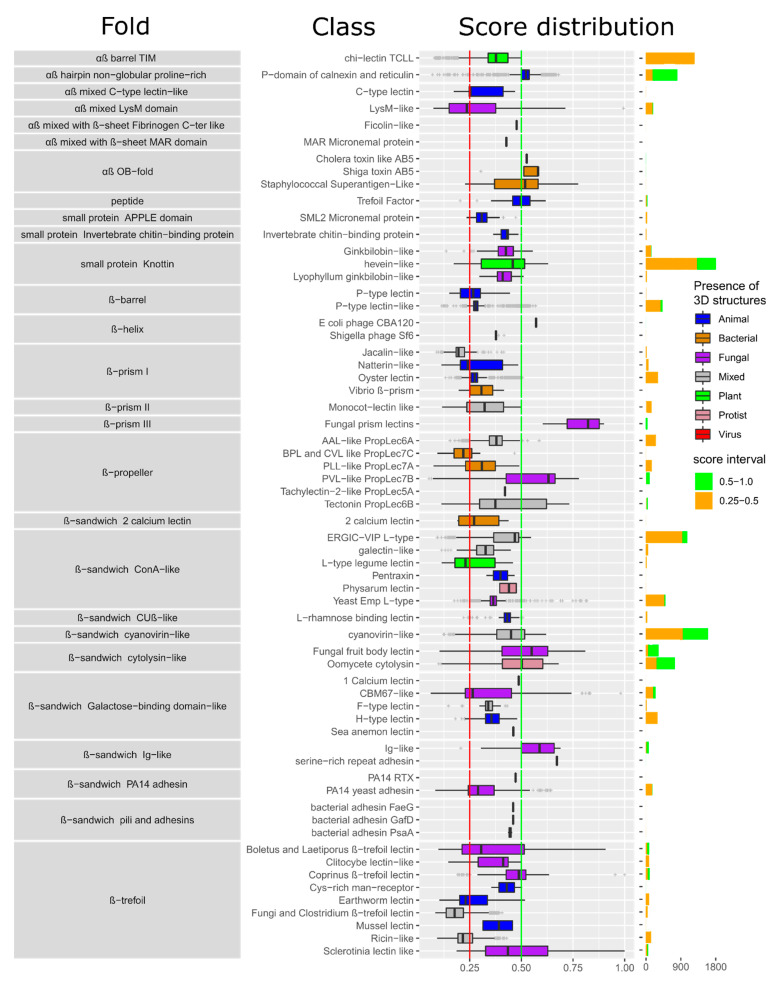
Distribution of the predicted fungal lectins sorted according to their fold and class. Boxplots are colored according to the structural origin of the lectin class defined by 3D structures. The right panel bar chart represents the lectin distribution by class where green represents high confidence (score > 0.5), and orange represents medium confidence predictions (score > 0.25).

The MycoLec database contains 33,518 putative lectin sequences (28,691 with filter at 0.25 quality score). Fungal lectin prediction spreads the classification coverage from 12 to 27 folds and 20 to 63 classes (see Section 3.1 and Figure S2). The distribution of lectins by class, along with the score distribution and the structural origin is displayed in Figure 3, illustrating that many more classes are predicted in genomes than characterized in structural studies. Notably, lectins are systematically found in fungal genomes. In 43% (12,290) of cases, the predicted lectins belong to a lectin class having a fungal origin in Unilectin3D (i.e., created using fungal proteins but not restricted to it). Expectedly, the prediction score of lectins containing lectin motifs of fungal origin (Figure 3, purple) is high, with the exception of the LysM-like lectin class. Surprisingly, predicted lectins matching lectin class profiles of non-fungal origin in Unilectin3D (or in the annotation) such as, Shiga-like toxin, Oomycete cytolysin and trefoil factors tended to score high as well. 

### 3.4. Phylogenetic Distribution of Fungal Lectins

**Figure 4:**
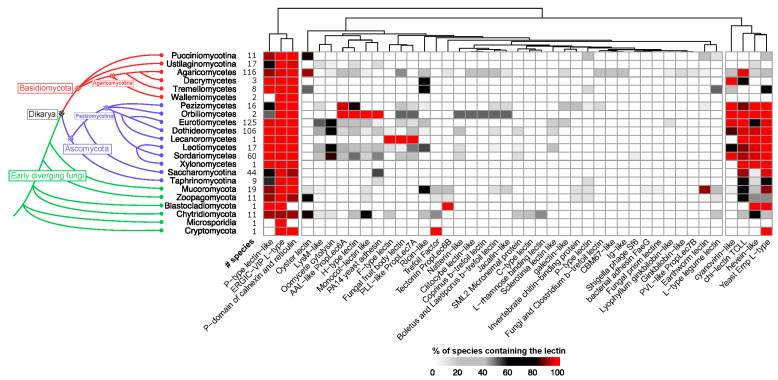
Distribution of predicted lectins by phyla and classes in MycoCosm genomes. Left, a phylogenetic tree showing the fungal divisions and classes as represented in the MycoCosm platform [32]. Right, clustering of lectin classes found in 582 species. Lectins with a similarity score >25% were used to compute the presence and abundance of the different lectin classes found in the MycoCosm genomes. The heatmap depicts lectin counts in each of the lectin classes according to the color scale displayed at the bottom of the figure.

The lectome composition was analyzed across the whole fungal kingdom (Figure S3). Figure 4 displays the clustering of predicted lectins in the different fungal sub-phyla and classes as displayed in the MycoCosm platform [32]. Strong variations of lectin catalogs are observed depending on the fungal lineage. Agaricomycetes display the largest variety, spanning more than 20 classes of lectins. The different classes of lectins are heterogeneously populated, with the strong dominance of a few classes. Ubiquitous lectins are in general house-keeping proteins, involved in quality control of glycoproteins. This is the case of calnexin and calreticulin, acting as chaperones by selective binding of a glucose residue on misfolded glycoproteins and recruitment of folding factors [31]. The P-type-like lectin is involved in misfolded glycoprotein ER-associated degradation (ERAD) in the cytosol. The carbohydrate recognition domain of p58/ERGIC-53 participates in glycoprotein export from the endoplasmic reticulum [33]. Other lectins are involved in non-self-recognition, such as TCLL, which is a degenerated chitinase (chitinase has an essential role in fungi) involved in antiviral activity. The oyster lectin also has an N-glycan (HMTGs) binding activity with microbicide activity.

Some lectin classes such as cyanovirin-like and fungal fruit body lectins were frequently identified within Agaricomycetes species. These families also show an expansion in specific species and genus, suggesting that the corresponding lectins undergo rapid evolution. 

The “house-keeping” lectins involved in quality control in the biosynthesis of glycoproteins are identified in all fungal classes, except Cryptomycota and Microsporidia. Cyanovirin-like, chi-lectin TCLL and Hevein and Yeast Emp L-type lectins are enriched in Ascomycota and Blastocladiomycota, but they are comparatively less present in Basidiomycota. H-type lectin and monocot lectin-like are more present in Orbiliomycetes (containing nematode-trapping fungi), while the Lecanoromycetes (lichen fungi) are enriched in F-type and Fruiting body lectins. 

### 3.5. Distribution of Lectin Classes According to the Nutrition Modes

**Figure 5:**
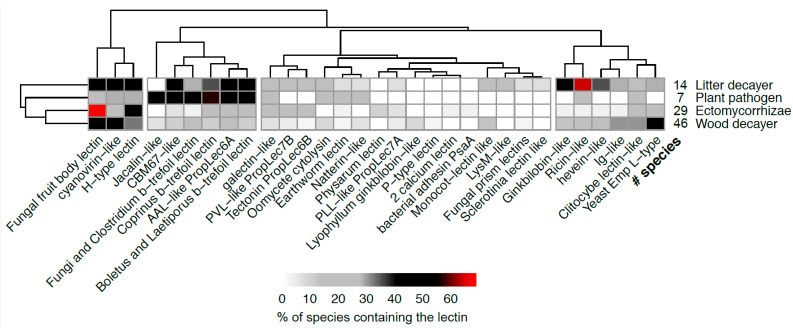
Occurrence of lectin classes in Agaricomycetes species grouped by lifestyle. Identified lectins are at least 25% similar to the reference. Ubiquitous lectins were filtered out. Only ecological groups represented by more than 5 species are represented.

As observed above, Agaricomycetes (Basidiomycota) display the most diverse lectin composition. Since these fungi adopt a large variety of lifestyles, their lectomes were analyzed as a function of ecology. After filtering out the ubiquitous lectins and those that are not present in Agaricomycetes (or as a single copy), the distribution of the 27 classes of interest was analyzed (Figure 5 and Table S2). Half of the species display three or fewer classes of lectins (48/107), but five of them have more than 10 classes.

The larger variety of lectin classes is observed in litter decayers with more than 10 different classes for some species, spanning more than 1000 genes, coding for putative lectins. The wood decayers also display a larger variety of lectins than the pathogenic or mycorrhizal fungi. Endophyte shows the smallest number of lectins, although the sample is very limited in this study (Table S2). 

Large variations across the lectomes of Agaricomycetes species can be observed but with no apparent correlation with the nutrition modes. However, decayers tend to display larger varieties of lectin classes (≥23) than symbionts and pathogens (≤17). The fungal fruit body lectins are prevalent, occurring in 50% of the investigated genomes. Cyanovirin-like proteins, 6-blades β-propellers and several β-trefoil lectin classes such as ricin-like and Coprinus-type are also found in many species. Cyanovirin and β-trefoil are widely distributed in eukaryotes and prokaryotes, while fungal fruit body lectins, also referred as actinoporin-type lectins [11] are highly specific to fungi, in both Ascomycota and Basidiomycota, with the only other occurrence in primitive plants such as Bryophyta and Hepatophyta.

Our analysis (Figure 4 and Figure 5) highlights the identification of two lectin classes not yet characterized experimentally in fungi. The GalNAc-specific H-type lectin is involved in self/non-self-recognition in invertebrates, and was first structurally characterized in snail eggs [36], but it was never isolated in fungi. A recent review on H-type lectins identified the corresponding sequence in the genome of several Agaricomycetes, including mycorrhizae *Tulaneslla calosporra* and wood decayer *Exidia glandulosa* [37]. Our study reveals a 30% occurrence of the GalNAc-specific H-type lectins in the investigated genomes. Similarly, a jacalin-like lectin was recently reported in *Grifola frondosa* (syn. *Polyporus frondosus*) [38], and the present work confirms the occurrence of this class in several other Agaricomycetes.

### 3.6. Prediction of the Laccaria Bicolor Lectome

*Laccaria* sp. are fungi pertaining to the Basidiomycota phylum and the Agaricomycetes class. It forms ECM with a wide range of trees. *L. bicolor* is one of the rare ectomycorrhizal fungus that can be genetically manipulated by using RNAi silencing or gene overexpression. This potentially allows investigation of the role of candidate lectin genes in further functional analyses. Only one strain for *L. bicolor* was publicly accessible at the time of this study and integrated in our dataset. A search with a 0.25 cutoff identified 13 different classes of lectins in the genome (Table S3). 

**Figure 6:**
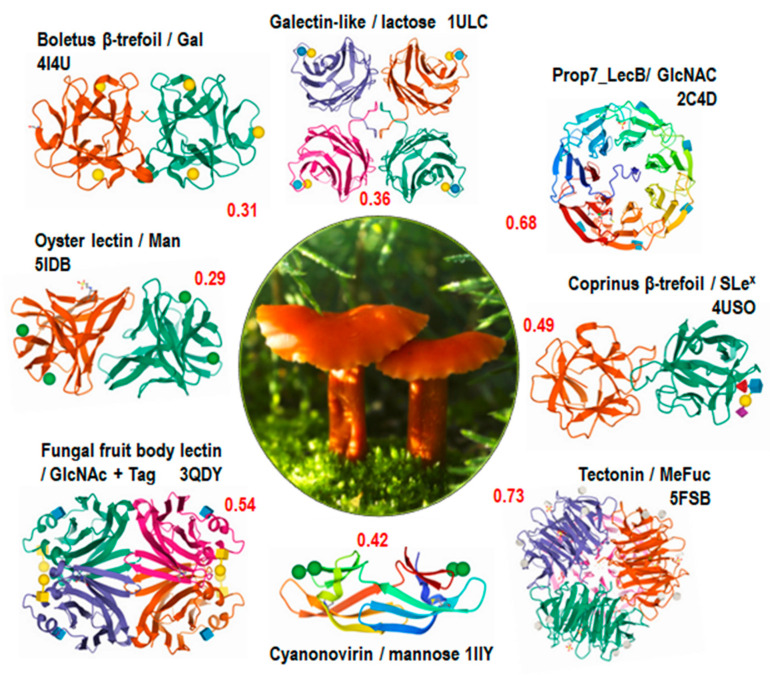
Part of the predicted lectome of *Laccaria bicolor*, with scores indicated in red. Only lectin classes with strong score prediction and conserved binding sites were selected and represented using Pymol (https://pymol.org/). Representative images for each class are from the PDB (www.rcsb.org) [35] and are generally from other species, except the tectonin, which is from *L. bicolor*.

Filtering out house-keeping lectins and poorly scored predictions left eight lectins that are displayed in Figure 6. Two of them are proteins found in many organisms: Cyanovirin and Oyster lectin. Cyanovyrin, originally from cyanobacteria [39] has been identified in plants and fungi, but its function is not elucidated. Oyster lectin has been recently found in bivalves [40] and is predicted to occur in many organisms, where its mannose-binding function is not yet clarified. The six other lectins are well characterized in different fungi where they play a role in defense against pathogens. Tectonin, structurally characterized in *L. bicolor* [41], binds to methylated sugars that are present in nematodes [42], while the galectin-like lectin CGL2 from *Coprinopsis cinerea* inhibits the development of nematodes [43]. Interestingly, the *L. bicolor* lectome contains a full panel of fungi-related lectins.

*L. bicolor* is a model for studying the establishment of ECM with the *Populus tremula x alba* tree [44]. This justified checking whether its many lectins are involved in this process. Transcriptional regulation of lectin genes has been investigated in 14 mycorrhizal associations, and RNAseq experiments were undertaken to differentiate gene expression with and without contact with a compatible plant [27,45,46,47,48] (Table S1). For most lectin genes, no variation in expression level was observed upon contact with the host plant, suggesting that only a restricted set of lectins might be involved in mycorrhization (Figure S4). Interestingly, several *L. bicolor* lectin genes, i.e., tectonin, β-trefoil lectins, cyanovirin and fungal fruiting body lectins, are upregulated during the mycorrhization (Figure S5). However, this observation is not valid in other fungi/tree associations, and in some cases, lectin gene transcription is downregulated during mycorrhization. Nonetheless, the differential expression of lectin genes in fungi interacting with trees compared with the isolated condition may show the importance of specific lectins in some symbiotic host interactions. Further studies are necessary to assess the role of lectins in the establishment of symbiosis. 

## 4. Discussion and Conclusions

The aim of this project was to explore lectin composition and abundance across and in the context of the fungal kingdom. A large panel of 33,518 fungal lectins belonging to 63 distinct lectin classes were predicted in 1419 genomes from the MycoCosm database. This collection ranked by a quality score can be accessed, searched and browsed in MycoLec, an online interactive database available at unilectin.eu/mycolec. Each predicted lectin is available together with the identified lectin domain in which the binding site can be compared with a reference motif. Significant differences in the lectin content (lectomes) of translated genomes support the distinction between fungal taxonomic classes. Moreover, lectin occurrence could be correlated with ecological information available in some of the fungal species considered in this study.

Based on the Agaricomycete fungal class, we showed that lectomes vary with lifestyle and that saprophytic fungi living on decaying wood and litters have a larger variety of lectins than symbionts and pathogens. However, an in-depth statistical analysis is necessary to demonstrate the association of lectins with specific ecological traits. Previous analysis of glycoside hydrolases in the Agaricales order [49] led us to reconstruct the history of the corresponding gene family and investigate the expansion and contraction events. Such a study of lectin evolution would be challenging because of the observed gene dispersion within the dataset.

*Laccaria bicolor*, a model fungus for studying ECM, includes interesting, predicted lectins with distinct folds and these proteins are upregulated in the presence of the associated host plant. This provides a promising lead for further analysis.

Overall, the present study opens up new prospects for appraising the broad diversity of fungal lectomes. Even though a large number of fungal lectins were already identified, we provide the tools to better appreciate the extent of this repertoire. We show that some lectin classes previously identified in invertebrates (H-lectins, oyster lectins) or in plants (jacalin, Ginkbilobin-like) turn out to be widely distributed in Agaricomycetes. Some of these new lectins could have useful application in biotechnology or as anti-viral compounds [50], and the MycoLec database is readily available to be mined for novel lectins.

## Data Availability

All data are available from the Unilectin.eu database.

## Supplementary Materials

The following are available online at https://www.mdpi.com/article/10.3390/jof7060453/s1, Figure S1: Distribution of lectin folds and classes of fungal lectin with 3D structures in Unilectin3D database, Figure S2: Distribution of folds (left) and classes (right) of predicted lectin sequences in MycoLec, Figure S3: Distribution of predicted lectins by species in MycoLec, Figure S4: Impact of mycorrhization of 14 fungal strains with their corresponding host plant on lectin expression, Figure S5: Differential expression of lectins within mycorrhizal fungi upon plants interaction, Table S1: Fungal species investigated in the exploration of the lectins induced and repressed during their mycorrhization with a compatible plant host, Table S2: Lectin content in the predicted proteomes of the Agaricomycetes fungal class sorted by ecological niche, Table S3: Details of lectins identified in the genome of *Laccaria bicolor*.

## References

[1] Naranjo-Ortiz M.A., Gabaldón T. (2019). Fungal evolution: Major ecological adaptations and evolutionary transitions. Biol. Rev. Camb. Philos. Soc..

[2] Richards T.A., Leonard G., Mahé F., Del Campo J., Romac S., Jones M.D.M., Maguire F., Dunthorn M., De Vargas C., Massana R. (2015). Molecular diversity and distribution of marine fungi across 130 European environmental samples. Proc. R. Soc. Lond. B Biol. Sci..

[3] Stajich J.E. (2017). Fungal Genomes and Insights into the Evolution of the Kingdom. Microbiol. Spectr..

[4] Fischer M.S., Glass N.L. (2019). Communicate and Fuse: How Filamentous Fungi Establish and Maintain an Interconnected Mycelial Network. Front. Microbiol..

[5] Lis H., Sharon N. (1998). Lectins: Carbohydrate-specific proteins that mediate cellular recognition. Chem. Rev..

[6] Goldstein I.J., Winter H.C., Kamerling J.P. (2007). Mushroom Lectins. Comprehensive Glycoscience.

[7] Guillot J., Konska G. (1997). Lectins in higher fungi. Biochem. Syst. Ecol..

[8] Nordbring-Herz B., Chet I., Mirelman D. (1986). Fungal lectins and agglutinins. Microbial Lectins and Agglutinin: Properties and Biological Activity.

[9] Pemberton R.T. (1994). Agglutinins (lectins) from some british higher fungi. Mycol. Res..

[10] Singh R.S., Bhari R., Kaur H.P. (2011). Characteristics of yeast lectins and their role in cell-cell interactions. Biotechnol. Adv..

[11] Sabotič J., Ohm R.A., Künzler M. (2016). Entomotoxic and nematotoxic lectins and protease inhibitors from fungal fruiting bodies. Appl. Microbiol. Biotechnol..

[12] Goossens K., Willaert R. (2010). Flocculation protein structure and cell-cell adhesion mechanism in Saccharomyces cerevisiae. Biotechnol. Lett..

[13] Reynolds T.B. (2018). Going with the Flo: The Role of Flo11-Dependent and Independent Interactions in Yeast Mat Formation. J. Fungi.

[14] Willaert R.G. (2018). Adhesins of Yeasts: Protein Structure and Interactions. J. Fungi.

[15] Houser J., Komarek J., Kostlanova N., Cioci G., Varrot A., Kerr S.C., Lahmann M., Balloy V., Fahy J.V., Chignard M. (2013). A soluble fucose-specific lectin from *Aspergillus fumigatus* conidia—Structure, specificity and possible role in fungal pathogenicity. PLoS ONE.

[16] Richard N., Marti L., Varrot A., Guillot L., Guitard J., Hennequin C., Imberty A., Corvol H., Chignard M., Balloy V. (2018). Human Bronchial Epithelial Cells Inhibit Aspergillus fumigatus Germination of Extracellular Conidia via FleA Recognition. Sci. Rep..

[17] Giollant M., Guillot J., Damez M., Dusser M., Didier P., Didier E. (1993). Characterization of a Lectin from Lactarius deterrimus (Research on the Possible Involvement of the Fungal Lectin in Recognition between Mushroom and Spruce during the Early Stages of Mycorrhizae Formation). Plant Physiol..

[18] Díaz E.M., Vicente-Manzanares M., Sacristan M., Vicente C., Legaz M.-E. (2011). Fungal lectin of Peltigera canina induces chemotropism of compatible Nostoc cells by constriction-relaxation pulses of cyanobiont cytoskeleton. Plant Signal. Behav..

[19] Singh R., Walia A. (2014). Characteristics of lichen lectins and their role in symbiosis. Symbiosis.

[20] Singh R.S., Walia A.K., Kennedy J.F. (2020). Mushroom lectins in biomedical research and development. Int. J. Biol. Macromol..

[21] Astrom E., Stal P., Zenlander R., Edenvik P., Alexandersson C., Haglund M., Ryden I., Pahlsson P. (2017). Reverse lectin ELISA for detecting fucosylated forms of alpha1-acid glycoprotein associated with hepatocellular carcinoma. PLoS ONE.

[22] Audfray A., Beldjoudi M., Breiman A., Hurbin A., Boos I., Unverzagt C., Bouras M., Lantuejoul S., Coll J.L., Varrot A. (2015). A recombinant fungal lectin for labeling truncated glycans on human cancer cells. PLoS ONE.

[23] Varrot A., Basheer S.M., Imberty A. (2013). Fungal lectins: Structure, function and potential applications. Curr. Opin. Struct. Biol..

[24] Bonnardel F., Mariethoz J., Salentin S., Robin X., Schroeder M., Pérez S., Lisacek F., Imberty A. (2019). UniLectin3D, a database of carbohydrate binding proteins with curated information on 3D structures and interacting ligands. Nucleic Acids Res..

[25] Bonnardel F., Mariethoz J., Perez S., Imberty A., Lisacek F. (2021). LectomeXplore, an update of UniLectin for the discovery of carbohydrate-binding proteins based on a new lectin classification. Nucleic Acids Res..

[26] Grigoriev I.V., Nikitin R., Haridas S., Kuo A., Ohm R., Otillar R., Riley R., Salamov A., Zhao X., Korzeniewski F. (2014). MycoCosm portal: Gearing up for 1000 fungal genomes. Nucleic Acids Res..

[27] Kohler A., Kuo A., Nagy L.G., Morin E., Barry K.W., Buscot F., Canbäck B., Choi C., Cichocki N., Clum A. (2015). Convergent losses of decay mechanisms and rapid turnover of symbiosis genes in mycorrhizal mutualists. Nat. Genet..

[28] Nguyen N.H., Song Z., Bates S.T., Branco S., Tedersoo L., Menke J., Schilling J.S., Kennedy P.G. (2016). FUNGuild: An open annotation tool for parsing fungal community datasets by ecological guild. Fungal Ecology.

[29] Edgar R.C. (2004). MUSCLE: Multiple sequence alignment with high accuracy and high throughput. Nucleic Acids Res..

[30] Potter S.C., Luciani A., Eddy S.R., Park Y., Lopez R., Finn R.D. (2018). HMMER web server: 2018 update. Nucleic Acids Res..

[31] Oliver J.D., van der Wal F.J., Bulleid N.J., High S. (1997). Interaction of the thiol-dependent reductase ERp57 with nascent glycoproteins. Science.

[32] Spatafora J.W., Aime M.C., Grigoriev I.V., Martin F., Stajich J.E., Blackwell M. (2017). The Fungal Tree of Life: From Molecular Systematics to Genome-Scale Phylogenies. The Fungal Kingdom.

[33] Velloso L.M., Svensson K., Lahtinen U., Schneider G., Pettersson R.F., Lindqvist Y. (2002). Expression, purification, refolding and crystallization of the carbohydrate-recognition domain of p58/ERGIC-53, an animal C-type lectin involved in export of glycoproteins from the endoplasmic reticulum. Acta Crystallogr. Sect. D Biol. Crystallogr..

[34] Notova S., Bonnardel F., Lisacek F., Varrot A., Imberty A. (2020). Structure and engineering of tandem repeat lectins. Curr. Opin. Struct. Biol..

[35] Burley S.K., Berman H.M., Christie C., Duarte J.M., Feng Z., Westbrook J., Young J., Zardecki C. (2018). RCSB Protein Data Bank: Sustaining a living digital data resource that enables breakthroughs in scientific research and biomedical education. Protein Sci..

[36] Sanchez J.F., Lescar J., Chazalet V., Audfray A., Gagnon J., Alvarez R., Breton C., Imberty A., Mitchell E.P. (2006). Biochemical and structural analysis of *Helix pomatia* agglutinin (HPA): A hexameric lectin with a novel fold. J. Biol. Chem..

[37] Pietrzyk-Brzezinska A.J., Bujacz A. (2020). H-type lectins—Structural characteristics and their applications in diagnostics, analytics and drug delivery. Int. J. Biol. Macromol..

[38] Nagata Y., Yamashita M., Honda H., Akabane J., Uehara K., Saito A., Sumisa F., Nishibori K., Oodaira Y. (2005). Characterization, occurrence, and molecular cloning of a lectin from Grifola frondosa: Jacalin-related lectin of fungal origin. Biosci. Biotechnol. Biochem..

[39] Bewley C.A. (2001). Solution structure of a cyanovirin-N:Man alpha 1-2Man alpha complex: Structural basis for high-affinity carbohydrate-mediated binding to gp120. Structure.

[40] Unno H., Matsuyama K., Tsuji Y., Goda S., Hiemori K., Tateno H., Hirabayashi J., Hatakeyama T. (2016). Identification, Characterization and X-ray Crystallographic Analysis of a Novel Type of Mannose-Specific Lectin CGL1 from the Pacific Oyster Crassostrea gigas. Sci. Rep..

[41] Sommer R., Makshakova O.N., Wohlschlager T., Hutin S., Marsh M., Titz A., Künzler M., Varrot A. (2018). Crystal Structures of Fungal Tectonin in Complex with O-Methylated Glycans Suggest Key Role in Innate Immune Defense. Structure.

[42] Wohlschlager T., Butschi A., Grassi P., Sutov G., Gauss R., Hauck D., Schmieder S.S., Knobel M., Titz A., Dell A. (2014). Methylated glycans as conserved targets of animal and fungal innate defense. Proc. Natl. Acad. Sci. USA.

[43] Butschi A., Titz A., Wälti M.A., Olieric V., Paschinger K., Nöbauer K., Guo X., Seeberger P.H., Wilson I.B.H., Aebi M. (2010). Caenorhabditis elegans N-glycan core beta-galactoside confers sensitivity towards nematotoxic fungal galectin CGL2. PLoS Pathog..

[44] Martin F., Selosse M.A. (2008). The Laccaria genome: A symbiont blueprint decoded. New Phytol..

[45] Martino E., Morin E., Grelet G.-A., Kuo A., Kohler A., Daghino S., Barry K.W., Cichocki N., Clum A., Dockter R.B. (2018). Comparative genomics and transcriptomics depict ericoid mycorrhizal fungi as versatile saprotrophs and plant mutualists. New Phytol..

[46] Miyauchi S., Kiss E., Kuo A., Drula E., Kohler A., Sánchez-García M., Morin E., Andreopoulos B., Barry K.W., Bonito G. (2020). Large-scale genome sequencing of mycorrhizal fungi provides insights into the early evolution of symbiotic traits. Nat. Commun..

[47] Murat C., Payen T., Noel B., Kuo A., Morin E., Chen J., Kohler A., Krizsán K., Balestrini R., Da Silva C. (2018). Pezizomycetes genomes reveal the molecular basis of ectomycorrhizal truffle lifestyle. Nat. Ecol. Evol..

[48] Peter M., Kohler A., Ohm R.A., Kuo A., Krützmann J., Morin E., Arend M., Barry K.W., Binder M., Choi C. (2016). Ectomycorrhizal ecology is imprinted in the genome of the dominant symbiotic fungus Cenococcum geophilum. Nat. Commun..

[49] Ruiz-Dueñas F.J., Barrasa J.M., Sánchez-García M., Camarero S., Miyauchi S., Serrano A., Linde D., Babiker R., Drula E., Ayuso-Fernández I. (2020). Genomic Analysis Enlightens Agaricales Lifestyle Evolution and Increasing Peroxidase Diversity. Mol. Biol. Evol..

[50] El-Maradny Y.A., El-Fakharany E.M., Abu-Serie M.M., Hashish M.H., Selim H.S. (2021). Lectins purified from medicinal and edible mushrooms: Insights into their antiviral activity against pathogenic viruses. Int. J. Biol. Macromol..

