# Supplementary material for: A Comprehensive Phylogenetic and Bioinformatics Survey of Lectins in the Fungal Kingdom
Source: J Fungi (Basel). 2021 Jun 7;7(6):453. doi: 10.3390/jof7060453 (PMC8227253; doi:10.3390/jof7060453)
Supplement: Supplementary file 1 [file jof-07-00453-s001.zip › jof-1193696-supplementary.pdf]

Supplementary data

A Comprehensive Phylogenetic and Bioinformatics Survey of Lectins in the Fungal kingdom

Annie Lebreton, François Bonnardel, Yu-Cheng Dai, Anne Imberty, Francis M. Martin, Frédérique Lisacek

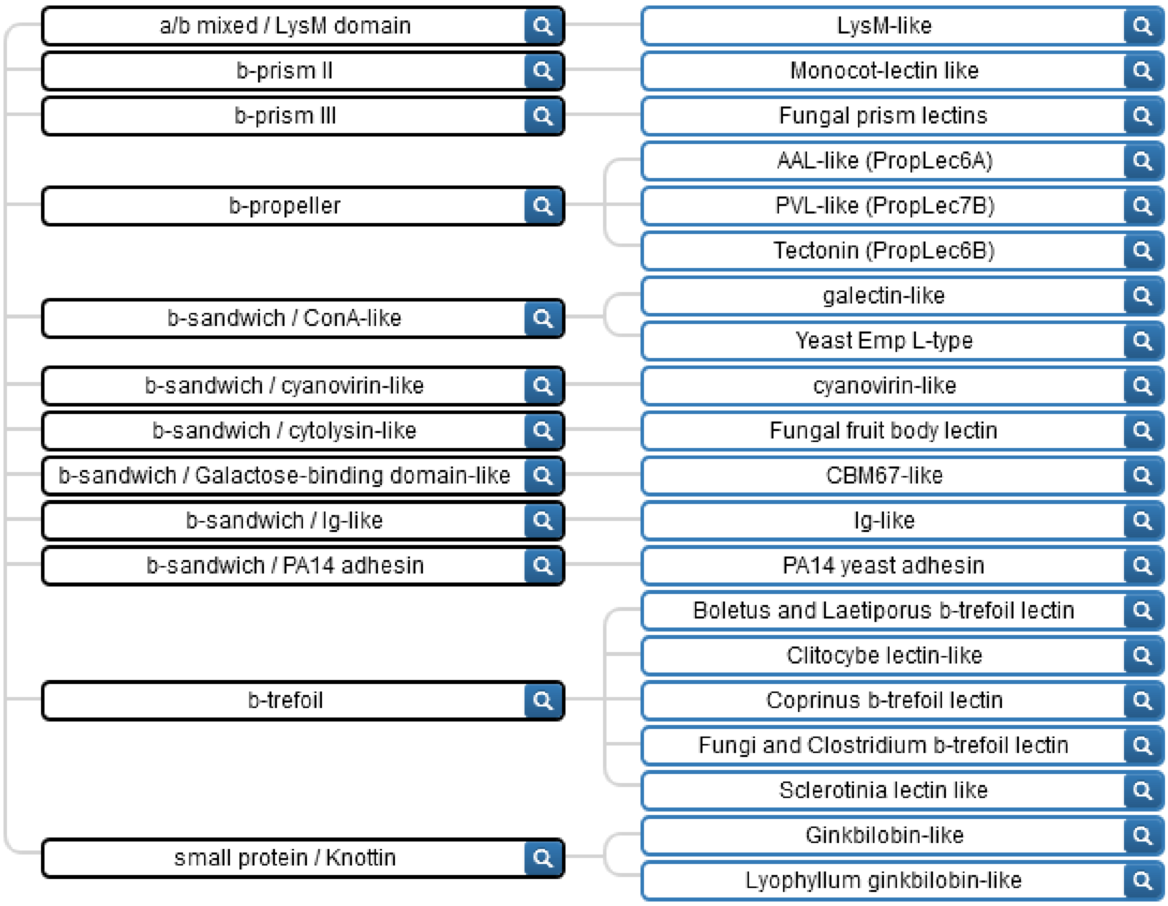

Figure S1: Distribution of lectin folds and classes of fungal lectin with 3D structures in Unilectin3D database.

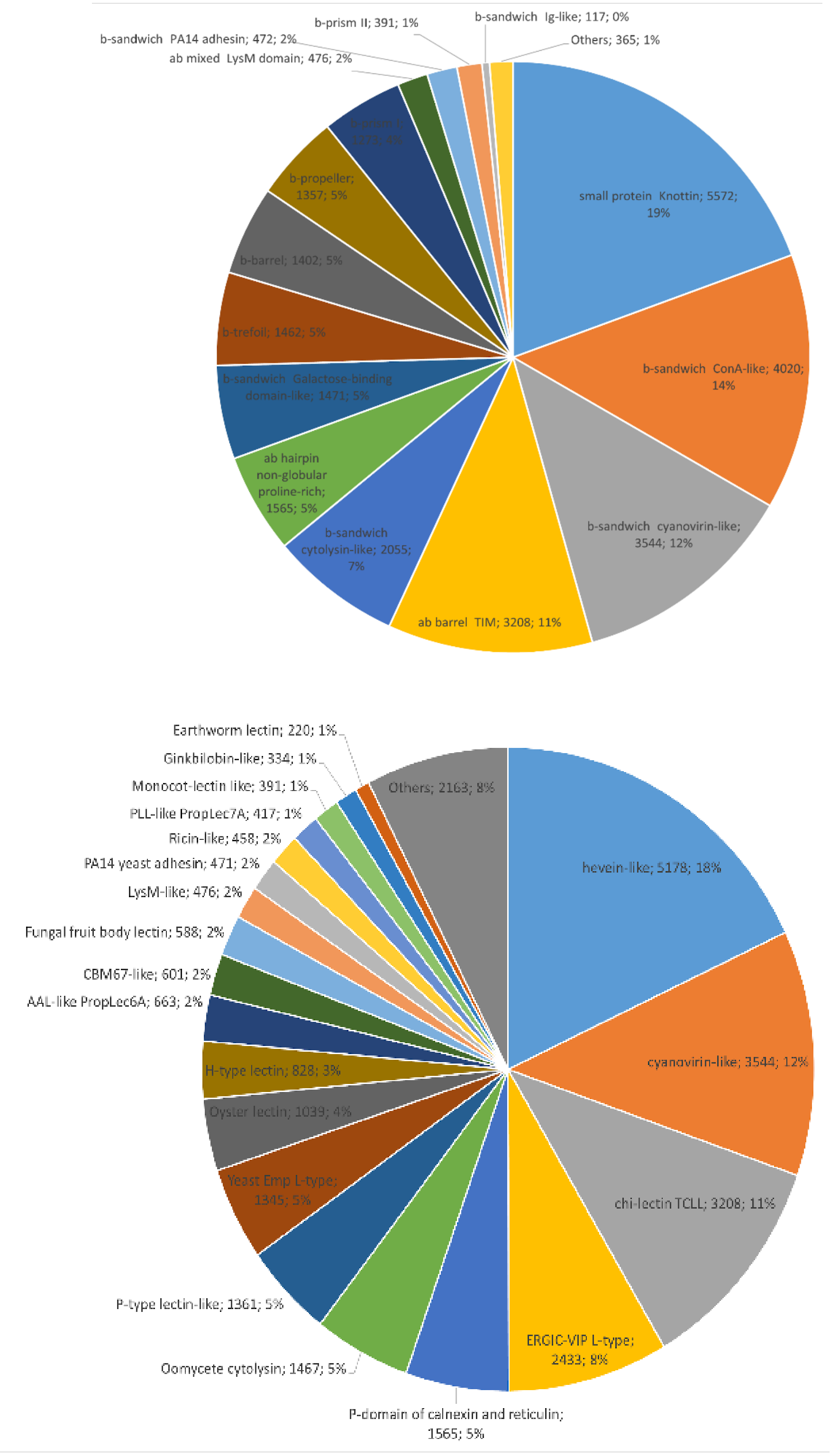

Figure S2: Distribution of folds (left) and classes (right) of predicted lectin sequences in MycoLec. Only lectin sequences with a similarity score > 25% were used.

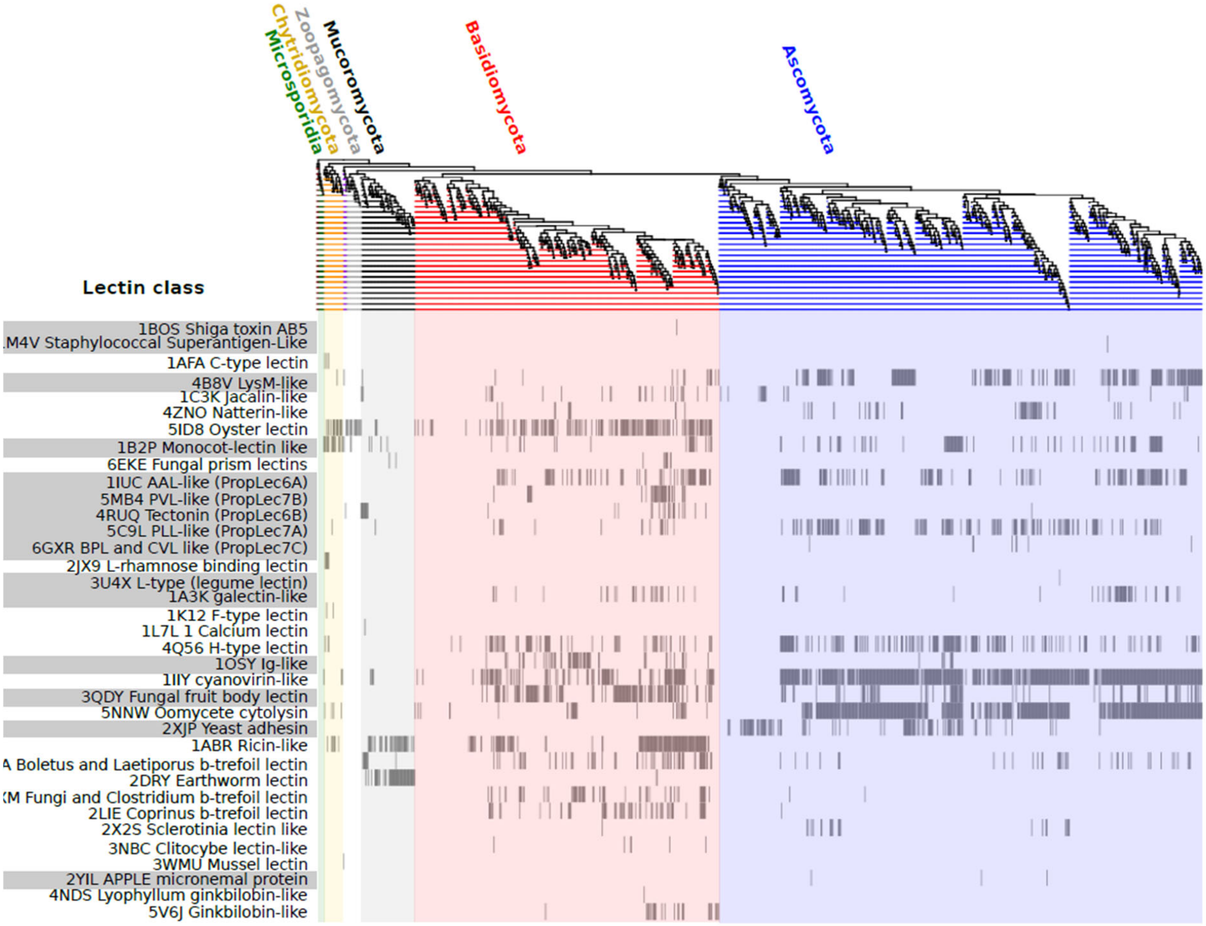

Figure S3: Distribution of predicted lectins by species in MycoLec. Each vertical line corresponds to a fungal strain organized according to their phylogenetic relationship as displayed by the tree. Left, Clustering of lectin classes. Lectins with a similarity score > 25% were used to detect the presence of the different lectin classes found in the MycoCosm genomes.

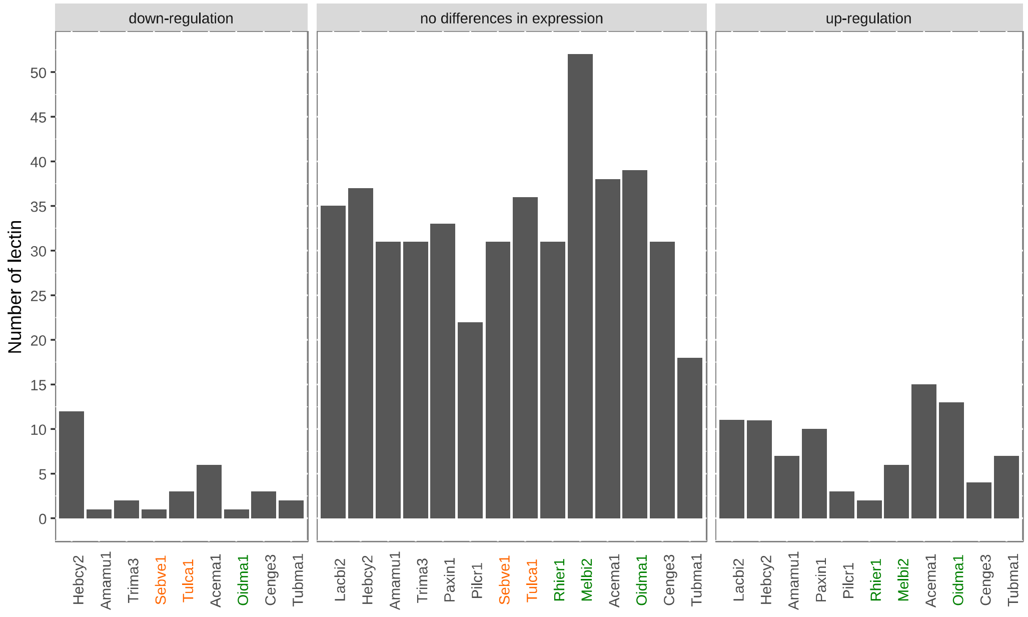

Figure S4: **Impact of mycorrhization of 14 fungal strains with their corresponding host plant on lectin expression.** Each bar corresponds to a strain annotated by a tag used in the MycoCosm database to refer to the specific strain and genomic assembly. Tags are colored according to the mycorrhizae type: grey ectomycorrhizae, green ericoid mycorrhizae, orange orchid mycorrhizae.

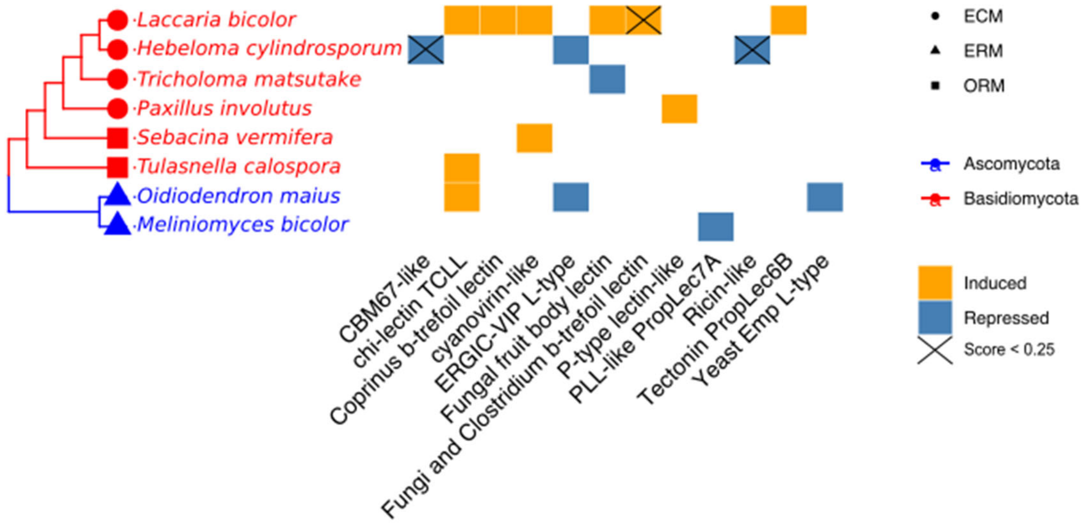

Figure S5: Differential expression of lectins within mycorrhizal fungi upon plants interaction. Lectins with invariable expression are not represented. Three species are ericoid mycorrhizae (ERM), two are orchids mycorrhizae (ORM) and the other are ectomycorrhizae (ECM).

Table S1: Fungal species investigated in the exploration of the lectins induced and repressed during their mycorrhization with a compatible plant host. ECM: ectomycorrhizae, ORM: orchids mycorrhizae, ERM: ericoid mycorrhizae

JGI ID	Fungal species	Plant host	Analysis Method	Mycorrhizae type	Ref
Amamu1	*Amanita muscaria*	*Populus tremula tremoloides*	CLC	ECM	[1]
Hebcy2	*Hebeloma cylindrosporum*	*Pinus pinaster*	CLC	ECM	[1]
Paxin1	*Paxillus involotus*	*Fagus sylvatica*	CLC	ECM	[1]
Pilcr1	*Piloderma croeus*	*Quercus robur*	CLC	ECM	[1]
Oidma1	*Oidiodendron maius*	*Vaccinium myrtillus*	CLC	ERM	[1]
Sebve1	*Sebacina vermifera*	*Arabidopsis thaliana*	CLC	ORM	[1]
Tulca1	*Tulasnella calosporra*	*Serapias vomeracea*	CLC	ORM	[1]
Melbi2	*Meliniomyces bicolor*	*Vaccinium myrtillus*	CLC	ERM	[2]
Rhier1	*Rhizoscyphus ericaceae*	*Vaccinium myrtillus*	CLC	ERM	[2]
Tubma1	*Tuber magnatum*	*Quercus robur*	CLC	ECM	[3]
Cenge3	*Cenococcum geophilum*	*Pinus sylvestris*	CLC	ECM	[4]
Acema1	*Acephala macrosclerotiorum*	*Pinus sylvestris*	HISAT/ DESeq2	ECM	[5]
Lacbi2	*Laccaria bicolor*	*Populus tremula x alba*	HISAT/DESeq2	ECM	[6]
Trima3	*Tricholoma matsutake*	*Pinus sylvestris*	CLC	ECM	[5]

Table S2: Lectin content in the predicted proteomes of the Agaricomycetes fungal class sorted by ecological niche.

	LysM-like	Jacalin-like	Natterin-like	Monocot-lectin like	Fungal prism lectins	AAL-like PropLec6A	PLL-like PropLec7A	PVL-like PropLec7B	Tectonin PropLec6B	galectin-like	Physarum lectin	Yeast Emp L-type	cyanovirin-like	Fungal fruit body lectin	Oomycete cytolysin	CBM67-like	H-type lectin	Ig-like	Boletus and Laetiporus b-trefoil	Clitocybe lectin-like	Coprinus b-trefoil lectin	Earthworm lectin	Fungi and Clostridium b-trefoil	Ricin-like	Ginkbilobin-like	hevein-like	Lyophyllum ginkbilobin-like		
**Endophyte**																													classes
Piriformospora indica	5													3														8	2
**Orchid mycorrhizae**																													0
Sebacina vermifera	1									1		1	2							2								7	5
Tulasnella calospora						1							1				6				3	1	1					13	6
**Ectomycorrhizae**																													0
Amanita muscaria																5	3	3				1						12	4
Amanita rubescens								3	6					1		11	6			4	1							32	7
Boletus edulis														9			3		3		5							20	4
Cantharellus anzutake									2				1															3	2
Cortinarius glaucopus						4		5	4			1					4		1					1		1		21	8
Gautieria morchelliformis							6					1		1			2											10	4
Gyrodon lividus													1	8			3											12	3
Hebeloma cylindrosporum							1	3	10	1			1	2											2			20	7
Hydnum rufescens																					3							3	1
Hysterangium stoloniferum						1						1					3											5	3
Laccaria amethystina								3	5	5	2			1							4							20	6
Laccaria bicolor								1	3	2	2		1	1					1		3							14	8
Lactarius quietus												1		4			11											16	3
Melanogaster broomeianus										2				2														4	2
Paxillus adelphus									1					3														4	2
Paxillus ammoniavirescens														5			3											8	2
Paxillus involutus											5			8			4											17	3
Piloderma croceum												1	1										1					3	3
Pisolithus tinctorius													4															4	1
Rhizopogon vesiculosus						1								1						4	4							10	4
Rhizopogon vinicolor						1								1						4	4							10	4
Russula ochroleuca														2			2											4	2
Scleroderma citrinum													20															20	1
Suillus brevipes						2								1						1								4	3
Suillus luteus						2								1														3	2
Thelephora ganbajun																			4									4	1
Thelephora terrestris						5								1														6	2
Tricholoma matsutake										1				1					1									3	3
Xerocomus badius/Imleria badia									2	2			1	2			2		1	1	8							19	8
**Grass decayer**																													0
Agaricus bisporus														1										2	2			5	3
Amanita thiersii										1		1				2											6	10	4
**Litter decayer**																													0
Ceriporiopsis (Gelatoporia)			4									1						1										6	3
Coprinellus micaceus								11					11				11		1					3	2	2		41	7
Coprinopsis cinerea								2		6							5			4	4			4				25	6
Coprinopsis marcescibilis								3		6		1												3	5			18	5
Fibulorhizoctonia sp.													2	20			7		1		1			1				32	6
Gymnopus androsaceus	1					2							5	1		1	3						1		6			20	8
Gymnopus luxurians				7								1	10	3		6						1		1	4			33	8
Leucoagaricus gongylophorus																								1				1	1
Marasmius fiardii				2		3			5					1						4				3				18	6
Mycena galopus	5					5				2			19		1	16	2	1	1	2	1			3	8	5		71	14
Polyporus arcularius															1			2					1			1		5	4
Psilocybe serbica					2	2			2				4	4		2	1	1	1		3		1		4	1		28	13
Pterula gracilis						1						1		2	1	1			1									7	6
Sphaerobolus stellatus						2	7						9	4		1			1		8		3	1	3	1		40	11
**Generalist decayer**																													0
Pleurotus ostreatus		2											3	2		4					1		1	1				14	7
Rhodosporidium toruloides												1																1	1
Serpula lacrymans						1							2								1		3					7	4
**Wood decayer**																													0
Auricularia subglabra													17	4			5	1		1		1					1	30	7
Auriculariopsis ampla						1						1												3				5	3
Bjerkandera adusta						6						1	1	7			2			1			1					19	7
Clavulina sp./Sistotrema													1	1						1			2					5	4

Coniophora olivacea												1	2	2							1							6	4
Coniophora puteana												1	1								1							3	3
Crucibulum laeve							1						1	3		3				1				2	9			20	7
Cylindrobasidium torrendii				1										1														2	2
Dentipellis sp.													1	3			3			1								8	4
Dichomitus squalens			3									1	1					3		4	1							13	6
Exidia glandulosa													20	2	1		1			1					3	3		31	7
Fibroporia radiculosa													1						1							1		3	3
Fistulina hepatica												1																1	1
Fomitopsis pinicola												1							1	2								4	3
Galerina marginata								2	2	1			6	1		7			1					1	6		1	28	10
Ganoderma sp.												1			1		1											3	3
Gloeophyllum trabeum													2															2	1
Heliocybe sulcata												1	1															2	2
Hypholoma sublateritium					1			1					5	1		3	1	1						1				14	8
Lentinellus vulpinus				1									1					1		1			3					7	5
Lentinula edodes		1																							1			2	2
Lentinus tigrinus															1			2					1					4	3
Neolentinus lepideus												1																1	1
Obba rivulosa			4	1								1						2										8	4
Omphalotus olearius																1		1										2	2
Peniophora sp.				1								2							2				5			2		12	5
Phanerochaete carnosa						2								2			6											10	3
Phanerochaete chrysosporium						6							1	2			1											10	4
Phlebia brevispora						1							1	1			2	2		1				2	7	2		19	9
Phlebia centrifuga													3	1			6											10	3
Phlebia radiata						3				1				1			8			1				7	9	2		32	8
Phlebiopsis gigantea						4							1	1			2			1								9	5
Plicaturopsis crispa												1											1					2	2
Pluteus cervinus		1				38				1				1		1		1						3		2		48	8
Polyporus brumalis															1			1					1			1		4	4
Postia placenta		1										1	3	4			2	2		7	3							23	8
Punctularia strigosozonata														3			1											4	2
Pycnoporus cinnabarinus												1											1					2	2
Ramaria rubella			1			1	3																	2				7	4
Schizophyllum commune				1		2						1												3				7	4
Schizopora paradoxa				2								1	6	1				1		1						1		13	7
Serpula himantioides						1							3								3		1					8	4
Stereum hirsutum												1		1			1											3	3
Trametes pubescens																		2									2	4	2
Trametes versicolor																		1									2	3	2
**Plant pathogen/Wood decayer**																													0
Armillaria cepistipes						2										16			1		4		2	1				26	6
Armillaria gallica						3										16			1		3		3	1				27	6
Fomitiporia mediterranea												1								2								3	2
Laetiporus sulphureus						1							1	1					2									5	4
**Plant pathogen**																													0
Armillaria mellea			1			1										1			3		5		2	1				14	7
Armillaria ostoyae						2										16			2		3		2					25	5
Armillaria solidipes		1				2										17			1		4		1					26	6
Heterobasidion annosum										1			2															3	2
Moniliophthora perniciosa		1													3													4	2
Rhizoctonia solani		1											8				3			4	3	3						22	6
Wolfiporia cocos														1			1											2	2
**Overall total**	**12**	**8**	**13**	**16**	**3**	**109**	**18**	**34**	**42**	**33**	**9**	**32**	**188**	**141**	**10**	**130**	**127**	**29**	**32**	**56**	**85**	**7**	**38**	**51**	**71**	**25**	**12**	**1331**	
**Number of species**	**4**	**7**	**5**	**8**	**2**	**32**	**5**	**10**	**11**	**15**	**3**	**31**	**44**	**53**	**8**	**20**	**36**	**19**	**22**	**25**	**27**	**5**	**22**	**24**	**15**	**14**	**5**	**107**	
% species	4%	7%	5%	7%	2%	30%	5%	9%	10%	14%	3%	29%	41%	50%	7%	19%	34%	18%	21%	23%	25%	5%	21%	22%	14%	13%	5%	100%	

Table S3 : Details of lectins identified in the genome of *Laccaria bicolor*

Lectin class	#	Mycocsm AC (score)	NCBI AC	Protein name
Tectonin PropLec6B	3	Lacbi2:399271 (0.73)<br>Lacbi2:399270 (0.67)<br>Lacbi2:322629 (0.27)	XP_001876432.1	tectonin 2
Coprinus β-trefoil lectin	3	Lacbi2:330799 (0.48)<br>Lacbi2:327918 (0.42)<br>Lacbi2:691792 (0.25)	XP_001885184.1	predicted protein [*Laccaria bicolor* S238N-H82]
Galectin like	2	Lacbi2:236913 (0.36)<br>Lacbi2:312069 (0.35)	XP_001883510.1	galectin [*Laccaria bicolor* S238N-H82]
Physarium lectin	2	Lacbi2:381649 (0.39)<br>Lacbi2:322629 (0.39)	XP_001875654.1	ricin-containing lipase tectonin-like
Oyster lectin	2	Lacbi2:585014 (0.28)<br>Lacbi2:448672 (0.27)	XP_001880964.1	predicted protein [*Laccaria bicolor* S238N-H82]
Fungal fruit body lectin	1	Lacbi2:185716 (0.54)	XP_001885326.1	predicted protein, partial
Boletus and Laetiporus β-trefoil lectin	1	Lacbi2:318163 (0.30)	XP_001879265.1	predicted protein [*Laccaria bicolor* S238N-H82]
Cyanovirin like	1	Lacbi2:327824 (0.42)	XP_001881773.1	predicted protein [*Laccaria bicolor* S238N-H82]
P domain of calnexin and reticulin	1	Lacbi2:399410 (0.50)	XP_001874124.1	calnexin [*Laccaria bicolor* S238N-H82]
Ergic vip L type	1	Lacbi2:399414 (0.48)	XP_001888824.1	ERGIC53, mannose lectin
P-type lectin like	1	Lacbi2:642707 (0.29)	XP_001874815.1	predicted protein [*Laccaria bicolor* S238N-H82]
PVL like PropLec7B	1	Lacbi2:692684 (0.67)	XP_001891161.1	predicted protein, partial

References

1. Kohler, A.; Kuo, A.; Nagy, L.G.; Morin, E.; Barry, K.W.; Buscot, F.; Canbäck, B.; Choi, C.; Cichocki, N.; Clum, A., et al. Convergent losses of decay mechanisms and rapid turnover of symbiosis genes in mycorrhizal mutualists. *Nat. Genet.* **2015**, *47*, 410-415, doi:10.1038/ng.3223.

2. Martino, E.; Morin, E.; Grelet, G.-A.; Kuo, A.; Kohler, A.; Daghino, S.; Barry, K.W.; Cichocki, N.; Clum, A.; Dockter, R.B., et al. Comparative genomics and transcriptomics depict ericoid mycorrhizal fungi as versatile saprotrophs and plant mutualists. *New Phytol.* **2018**, *217*, 1213-1229, doi:10.1111/nph.14974.

3. Murat, C.; Payen, T.; Noel, B.; Kuo, A.; Morin, E.; Chen, J.; Kohler, A.; Krizsán, K.; Balestrini, R.; Da Silva, C., et al. Pezizomycetes genomes reveal the molecular basis of ectomycorrhizal truffle lifestyle. *Nat. Ecol. Evol.* **2018**, *2*, 1956-1965, doi:10.1038/s41559-018-0710-4.

4. Peter, M.; Kohler, A.; Ohm, R.A.; Kuo, A.; Krützmann, J.; Morin, E.; Arend, M.; Barry, K.W.; Binder, M.; Choi, C., et al. Ectomycorrhizal ecology is imprinted in the genome of the dominant symbiotic fungus Cenococcum geophilum. *Nat. Commun.* **2016**, *7*, 12662, doi:10.1038/ncomms12662.

5. Miyauchi, S.; Kiss, E.; Kuo, A.; Drula, E.; Kohler, A.; Sánchez-García, M.; Morin, E.; Andreopoulos, B.; Barry, K.W.; Bonito, G., et al. Large-scale genome sequencing of mycorrhizal fungi provides insights into the early evolution of symbiotic traits. *Nat. Commun.* **2020**, *11*, 5125, doi:10.1038/s41467-020-18795-w.

6. Basso, V.; Kohler, A.; Miyauchi, S.; Singan, V.; Guinet, F.; Simura, J.; Novak, O.; Barry, K.W.; Amirebrahimi, M.; Block, J., et al. An ectomycorrhizal fungus alters sensitivity to jasmonate, salicylate, gibberellin, and ethylene in host roots. *Plant Cell Environ* **2020**, *43*, 1047-1068, doi:10.1111/pce.13702.
